# Health poverty alleviation in China from the perspective of historical institutionalism: policy changes and driving factors

**DOI:** 10.3389/fpubh.2023.1265588

**Published:** 2024-01-17

**Authors:** Li Xu, Xiaojian You, Yinan Cui, Jiali You

**Affiliations:** School of Marxist, Southwest University of Science and Technology, Mianyang, China

**Keywords:** health poverty alleviation, policy changes, historical review, driving force, framework of historical institutionalism

## Abstract

Health poverty alleviation is an effective tool for improving the living quality and developmental conditions of impoverished populations. Since 1978, China has been actively implementing health poverty alleviation projects, resulting in a more robust rural healthcare service network and increased convenience for the local population to access medical treatment. However, it is crucial to acknowledge that China still faces a complex situation with the simultaneous existence of multiple disease threats and the interweaving of various health influencing factors. Ongoing risks of emerging infectious diseases persist, and some previously controlled or eliminated infectious diseases are at risk of resurgence. The incidence of chronic diseases is on the rise and exhibits a trend toward affecting younger populations. Therefore, examining the successful experiences of China's health poverty alleviation over the past 40 years becomes a critically important issue. The study focuses on China's health poverty alleviation policies, employing historical institutionalism as a theoretical perspective to analyze the historical changes and evolutionary logic of health poverty alleviation policies. A historical institutionalist analytical framework for health poverty alleviation policies is constructed. The research findings reveal that China's health poverty alleviation policy has undergone three distinct periods since 1978: the initial phase (1978–2000), the exploratory phase (2000–2012), and the stable development phase (2013–present). At the macro level, the political, economic, and social contexts of different periods have influenced the evolution of health poverty alleviation policies. On the meso level, coordination effects and adaptive expectations have had an impact on China's health poverty alleviation policy. At the micro level, various actors, including the central government, local governments at different levels, social forces, and impoverished communities, interact during the evolution of health poverty alleviation policies. This paper summarizes the theoretical aspects of China's health poverty alleviation policy experience. The research conclusions, viewed through the lens of historical institutionalism, offer practical insights into the evolution of government policies. This provides directional guidance for enhancing health poverty alleviation projects.

## 1 Introduction

**Figure 1:**
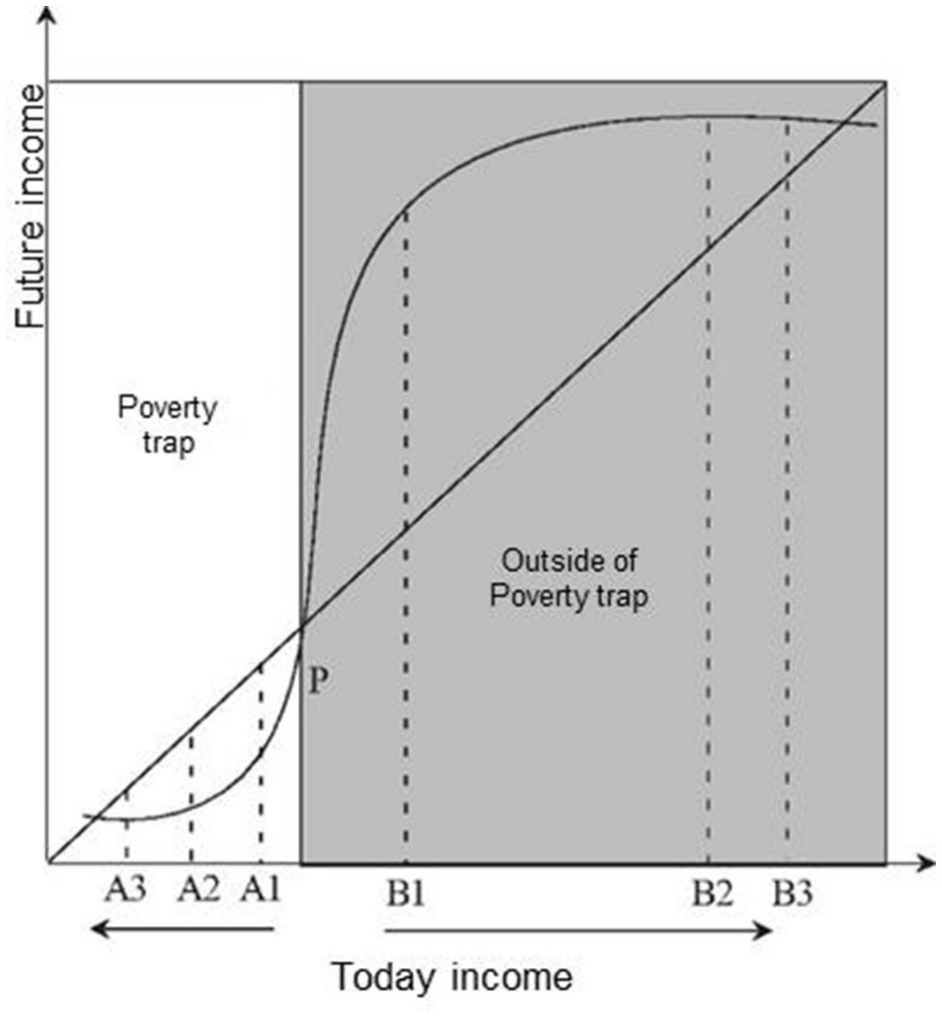
Poverty trap hypothesis.

Health poverty refers to the loss of access to health services and the means of life development caused by insufficient economic affordability, which is ultimately manifested in a vicious circle of declining health and increasing poverty. Health is closely associated with poverty. While poverty is liable to breeding diseases, poor health opens a window to poverty. Poor people are hard to avoid a fall into so-called vicious circle as “poverty → disease → poverty” (1). Looking on the “poverty trap” hypothesis, the X-axis is the income of the population today, and the Y-axis is the income of the future. The diagonal line indicates that today's income is equal to tomorrow's income, plus the S-shaped curve that shows the source of the “poverty trap”. Note that the left strip of point P displays a “poverty trap.” The curve is below the diagonal, i.e., the future income goes lower than today's income. Alternatively, people in this area may become poorer and poorer over time, from A1 → A2 → A3, and eventually fall into endless poverty. In the right strip of point P, the curve is higher than the diagonal, which means that the income in the future goes higher than the income today, and the people in this area may get richer. From B1 to B2, B3, the curve gets flattening finally as shown in Figure 1.

To prevent the poor from falling into the “poverty trap” due to disease, the poor must be given the opportunity to cross point P, and further break the cycle of poverty and disease through health poverty alleviation. The critical link to health poverty alleviation is to help the poor reduce medical expenditures while improving health by increasing labor productivity and reducing household burden rates by raising incomes. Health poverty alleviation aims to promote the development of medical and health businesses in poverty-stricken areas through preferential support in medical care, medical security, health education, and basic public health services. Once the overall level of medical treatment for the poor people is improved, the quality of their life and development conditions would go up to a higher level (2). In 2018, China implemented financial poverty alleviation, industrial poverty alleviation, employment poverty alleviation, education poverty alleviation, and health poverty alleviation. The income of rural residents in poverty-stricken areas has increased rapidly, and the results of poverty alleviation have become increasingly remarkable (3). In the first quarter, the per capita disposable income of rural residents in poverty-stricken areas increased by 11% year-on-year. Excluding price factors, the actual increase was 8.8%, 2% points faster than the per capita disposable income of rural residents nationwide (4).

Sadik-Zada analyzed the nexus between natural resource abundance and economic growth from the lens of socioeconomic dualism. From the perspective of development economics, the theoretical level that savings behavior of workers in the nascent modern sector has the capacity of partial mitigation the allocative inefficiencies and the negative modernization effects that emanate from distributional bargaining behavior of the politically and economically powerful elite (5).

In fact, since 1978, the Chinese government has vigorously implemented the health poverty alleviation policy and promoted the development of the Healthy China Project. The journey consists of three phases:

### 1.1 The early stage (1978–2000)

In the early days of reform and opening-up, while effectively solving the problem of food and clothing for the people in poor areas, the state also began to consider solving the problem of the extreme shortage of basic medical and health resources for the extremely poor people. In December 1981, as pointed out in the Notice on Conscientiously Doing a Good Job in Helping Poor Rural Households: “Local health departments shall actively help poor households prevent and treat diseases, and reduce certain fees ... These practices may be gradually promoted by various localities in light of their own actual conditions,” which actually underlined the important role of medical and health relief in poverty alleviation (6). The Directive on Reforming and Strengthening Rural Medical and Health Work stated that “The medical and health work shall have the focus placed on rural areas, and necessary support shall be given especially for the construction of health services in poor areas.” Health poverty alleviation established an effective way for the masses to obtain medical assistance, and determined the effective connection between the government and the poor people. More than an essential part of poverty alleviation relief, health poverty alleviation has become a key work in the field of medicine and health in China.

### 1.2 The exploration stage (2000–2012)

After entering the twenty-first century, while the overall economic development was witnessed in this country, the problems of regional medical and health resources were also listed on the agenda. During this period, the national policies were mainly focused on solving the problem of equalization of rural health resources. In 2002, the government issued the Decision on Further Strengthening Rural Health Work. In 2009, the Opinions on Deepening the Reform of the Medical and Health System was issued. The two policies represented the establishment of a rural medical security system and the implementation of the rural medical assistance system, which were specially designed by the state to support the medical and health undertakings in poor areas. The state emphasizes the important role of the rural cooperative medical system in “ensuring that farmers have access to basic medical services and preventing poverty due to illness”. Specifically, the policy targets were located as the most difficult poor farmers and the most urgent medical expenditures. This measure specified the necessary special support for medical assistance in poor areas transferred by the central government.

### 1.3 The development stage (2013–present)

Since 2013, the national policy on health poverty alleviation has been gradually developed to a mature level as reflected in: First, improve the level of medical security and effectively reduce the burden of medical expenses on the rural poor. Second, continuously accelerate the construction of medical and health services in poverty-stricken areas. In poverty-stricken areas, projects such as rural order-oriented free medical student training, special post plans for general practitioners, and training of health personnel in the western region were implemented (7). Pilot projects for comprehensive training of health and family planning talents were carried out, and efforts were made to alleviate the shortage of talents that hindered the development of health and family planning in poor areas. Third, the counterpart support work of medical and health institutions was deepened. One thousand six hundred and forty-four tertiary hospitals across the country have been organized to establish counterpart support relationships with 3,945 county-level hospitals, of which 832 county-level hospitals in poor counties are basically assisted by urban tertiary hospitals. Fourth, work on the prevention and control of public health and disease has been continuously strengthened. Take public health action to prevent various diseases, including: childhood vaccination; established health records, free physical examinations for residents, and closer follow-up; intensified surveillance and prevention and control of infectious diseases; strengthened chronic disease management; free pre-pregnancy eugenic health examinations for women and children, folic acid supplementation for rural women to prevent neural tube defects, free hospital delivery for poor pregnant women, breast and cervical cancer screening for rural women, improvement of children's nutrition, screening for newborn diseases, etc., (8). In 2020, the screening rate of common diseases among women hit 86.6%, an increase of 25.4% points over 2010.

## 2 Literature review, research data, and analysis framework

### 2.1 Review of relevant research

Alam and Mahal proposed that poverty is a living condition people are unwilling to endure, signifying a lack of food, resources, opportunities, and even the loss of freedom (9). McIntyre et al. examined the current status of health inequality between the poor and non-poor, as well as the consequences of poverty and income inequality related to healthcare expenses. It is believed that poverty and poor health are interwoven, with poverty breeding ill health, and poor health, in turn, trapping people in poverty. Conversely, health can be described as a form of wealth, possessing significant instrumental value in poverty reduction (10).

Asfaw and Jütting, in their study in Senegal, found that health insurance can increase the utilization of healthcare services and reduce the incidence of poverty (11). Aryeetey et al., studying Ghana's National Health Insurance Scheme implemented since 2004, concluded that enrollment can reduce the incidence of poverty by 7.5% (12).

In recent years, domestic scholars have conducted research on health poverty alleviation, yielding a series of achievements. Liu and Tan from the perspective of sustainable development, analyzed the inherent roots of the vicious cycle of health poverty (13). Wang et al. quantitatively studied poverty alleviation policies from the perspective of policy tools (14). Zhai and Yan explored the role and realization logic of health poverty alleviation (15). Zhang and Zhang, taking a longitudinal analysis perspective of national governance, analyzed the logical evolution of health poverty alleviation policies from policy separation - positive incentive to policy separation - negative incentive, and from a single system to comprehensive system integration for poverty alleviation (16). China's health poverty alleviation work has achieved success in maintaining social fairness and justice as well as promoting the development of human capital. The poverty rate in China decreased from 10.2% at the end of 2012 to 0.6% in 2019. From a macro perspective, Ting et al. discussed the effectiveness of health poverty alleviation policies from the perspective of equal health rights (17). They argued that under the “Healthy China” strategy, health poverty alleviation, adhering to a people-centered approach and valuing rights as a guiding value, ensures the health rights of the impoverished population, achieving equality in people's health rights (17).

In summary, domestic and foreign scholars agree that there is a negative correlation between health and poverty. Foreign scholars pay more attention to the economic burden caused by various diseases, and also put forward corresponding poverty alleviation strategies for the adverse economic consequences caused, but there are few studies on the implementation and implementation effect of local health poverty alleviation. Since the health poverty alleviation policy was officially proposed in 2015, the policy has received great attention from domestic scholars. At present, the research on health poverty alleviation policy by domestic scholars has been relatively systematic and perfect, involving the historical evolution, implementation, implementation effect, dilemma and path optimization of the policy, which provides a reference for this study.

However, the existing studies also have the following shortcomings: First, they do not further reveal the historical core logic of the evolution of China's health poverty alleviation policy. Most of the existing studies summarize the process of health poverty alleviation too macro, and it is difficult to comprehensively summarize and show the whole process. Based on the text of the health poverty alleviation policy, this paper will summarize three stages, which will greatly enrich the existing research on the journey of health poverty alleviation in China. Second, as far as the mechanism of health poverty alleviation is concerned, the existing studies only explain the basic points and general directions around the role and realization logic of relative health poverty alleviation, but the responsibilities and interactions of different actors in China's health poverty alleviation governance mechanism have not been clearly defined, that is, how this mechanism plays a role has not been fully studied, let alone introduced an appropriate theoretical perspective. Based on the Chinese scenario and the analytical framework of historical institutionalism, this paper explains the characteristics of China's health poverty alleviation policies at different stages of development, focuses on the driving factors affecting health poverty alleviation policies, and analyzes the driving effects of economic development level, concept ideology, and departmental function setting on health poverty alleviation. Through research, we can reveal the deep code of China's health poverty alleviation governance, present the special laws behind the “China story” and the general knowledge contained in it, and provide historical experience and suggestions.

### 2.2 Research data, research methods, and analytical frameworks

**Figure 2:**
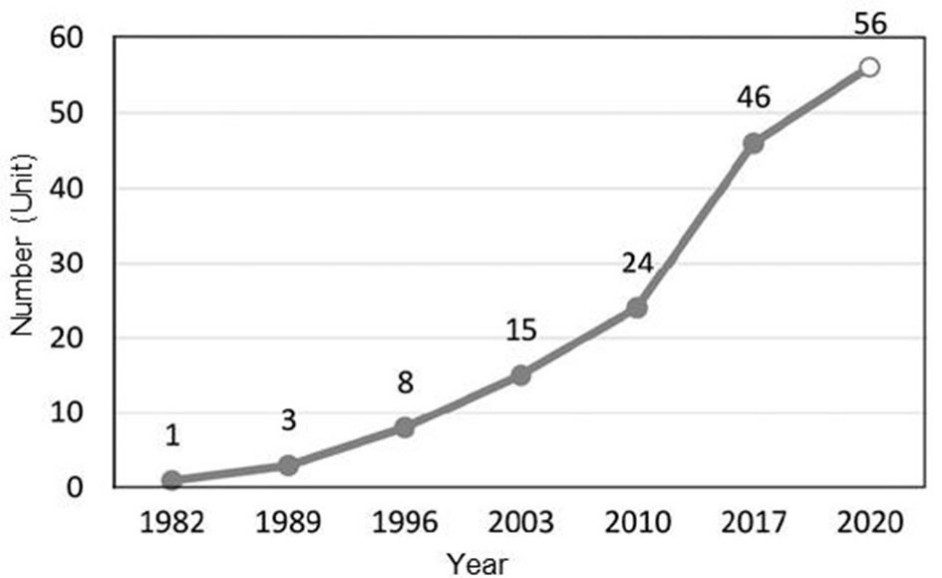
Cumulative distribution of government health poverty alleviation documents from 1982 to 2020.

The health poverty alleviation policies selected in this work are all from the official websites of the State Council Poverty Alleviation Office, the National Development and Reform Commission, the National Health Commission, the National Medical Security Administration, and the Ministry of Finance. As a concentrated embodiment of political system reform and economic and social development in the field of health poverty governance, the involved texts of health poverty alleviation policy must be able to clearly reflect the characteristics of the times and the evolution of health poverty alleviation policies (18) (Figure 2).

Historical institutionalism aims to explore the law of institutional change with time series analysis as its research method (19). This is a more explanatory theoretical analysis tool for studying institutional changes (20). More than macro factors, such a tool takes care of individual influences when analyzing institutional changes. Besides, path dependence, key nodes, contingency, initiative and concepts are also incorporated into the analysis of institutional changes. By constructing an all-round analytical basis of “macrostructure-meso system-micro actors,” historical institutionalism is able to establish a logical connection between the influencing factors of the system and the institutional changes. Looking into China's health poverty alleviation policy, all were born in a certain historical context. Their evolution and dynamic mechanism not only reflect the characteristics of institutional change itself, but also directly reflect the value orientation of actors. Therefore, the theoretical analysis framework of historical institutionalism may better explain the emergence, development and evolution of China's health poverty alleviation policies, and reveal the driving factors of policy changes.

**Figure 3:**
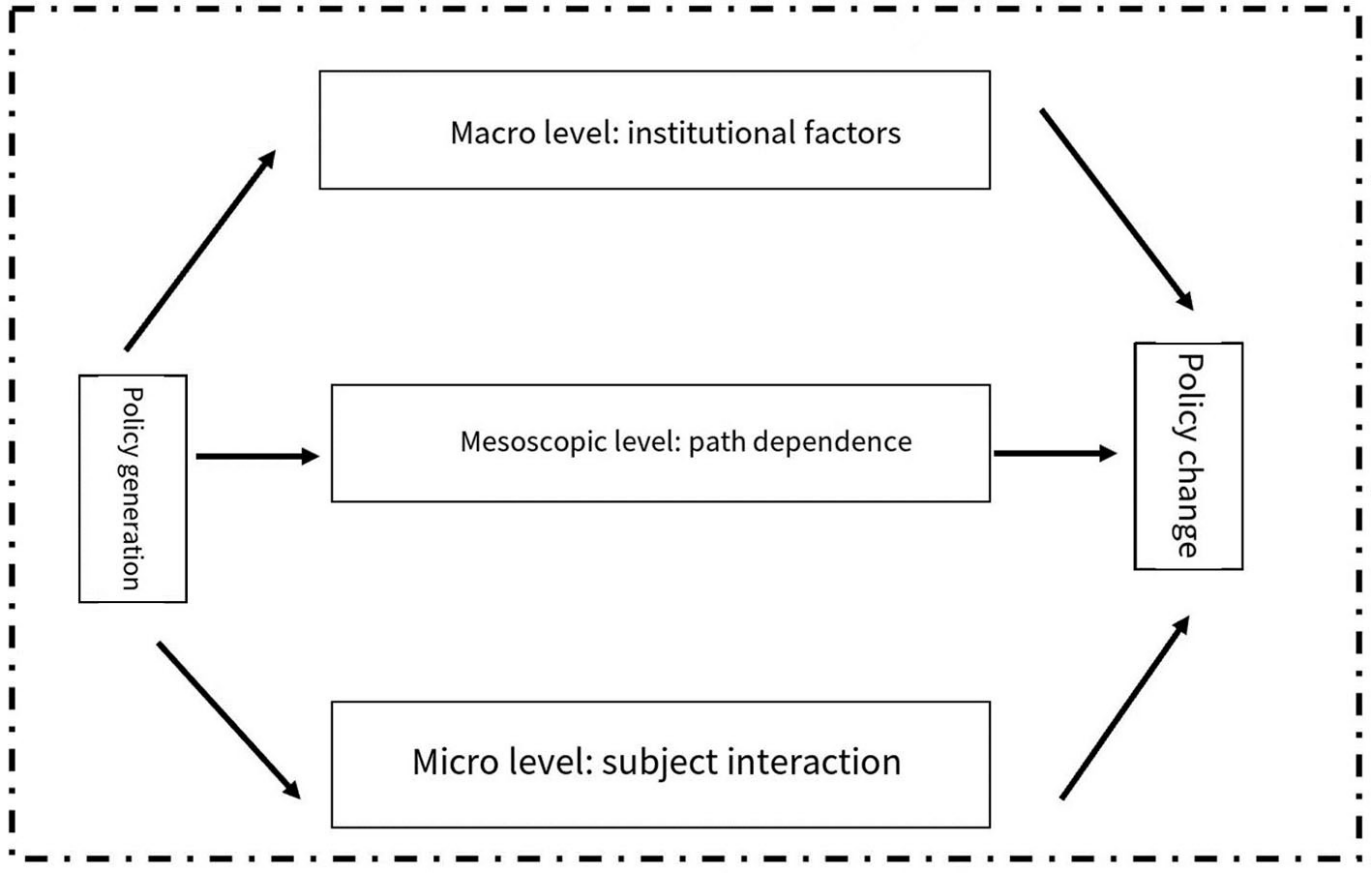
Theoretical analysis framework of the historical change of China's health poverty alleviation policy.

In this paper, historical institutionalism is introduced as a theoretical perspective to discuss the historical changes and evolution logic of health poverty alleviation policies. An analysis framework on historical institutionalism for health poverty alleviation policies is therefore constructed. Subsequently, the historical evolution of health poverty alleviation policies are combed to help analyze the dynamic factors affecting China's health poverty alleviation policies and explore the optimization path of future development. At the macro level, based on the process of poverty alleviation and development in China, this work investigated the political, economic and social backgrounds that may pose an impact on the changes of similar policies in different periods. At the level, from the perspective of path dependence, the impact of coordination effect and adaptive expectation on the change of China's health poverty alleviation policy are explored. At the micro level, in the process of health poverty alleviation policy change, the interaction between the central government, local governments at all levels, social forces and poor groups is analyzed. The three levels together constitute the basic logic and dynamic mechanism for the development and evolution of health poverty alleviation policies (Figure 3).

## 3 Research on the dynamic mechanism of China's health poverty alleviation policy

### 3.1 Macro level: institutional factors

#### 3.1.1 High-quality economic development

Lewis argues that the governments of many backward countries want to use surplus manpower for capital formation and that governments do influence the process of capital accumulation in many ways. “They will not make an effort unless they are guaranteed that the fruits of their efforts will be obtained by them or by them acknowledging the acquisition of their possession ... Many of the efforts of social reformers have been to change the system so that it can protect it.” In cooperative labor, where the worker is separated from property, the motivational factors of the worker are incentives, authority, and the distribution of income between labor and capital ownership (21). Since 1978, the Chinese government has encouraged enterprises to implement effective work incentive mechanisms, which has greatly promoted the improvement of enterprise production efficiency.

The current debate on whether China has arrived at the Lewis turning point highlights this difficulty. Since the 1978 economic reforms, China—in just three decades—has experienced a rapid and pronounced economic transformation from a planned to a market economy and from one based on agriculture to one emphasizing industry. The success of rural reform in the late 1970's and early 1980's greatly improved agricultural productivity and simultaneously released a tremendous amount of surplus labor from the land (22). As a result, a large number of laborers moved from the agricultural to the industrial and service sectors. For more than two decades following the economic reform, the supply of labor seemed to be unlimited, enabling China's manufacturing sector to maintain a comparative advantage in labor-intensive products. Fueled by this seemingly endless supply of cheap labor, many of China's manufactured goods became so competitive in the international market that China earned the name “the world's factory (23).”

The high-quality development of the Chinese economy fundamentally ensures health poverty alleviation. On one hand, economic growth implies more employment opportunities and higher income levels. Through sustained economic growth, individuals can secure more job opportunities, elevate their income levels, and thus escape poverty. In the assessment report of the World Health Organization's Commission on Macroeconomics and Health (CMH), economic output is considered a result of policy and institutional effects, and economic inputs include not only human capital and technological capabilities but also corporate capital, among others.

On the other hand, the enhancement of China's economic strength has comprehensively ensured basic healthcare for rural impoverished populations, successfully lifting nearly 10 million families out of poverty due to illness-induced impoverishment. A nationwide dynamic monitoring system to prevent a return to poverty due to illness has been established, conducting dynamic monitoring of populations prone to falling back into poverty. Various targeted assistance measures have been implemented, continuously increasing support in terms of policies, funds, and projects, and comprehensively enhancing the medical and health service capabilities in poverty-stricken areas. To address the issue of weak medical service capabilities in impoverished areas, China has implemented numerous targeted assistance measures. For instance, since 2016, Peking University First Hospital has dispatched over 300 experts to hospitals such as the Central Hospital of Lankao County in Henan Province, the People's Hospital of Linquan County in Anhui Province, and the People's Hospital of Yonghe County in Shanxi Province. They have assisted in more than 150 new technologies and projects, conducting ~2,000 surgeries to support the local medical services.

#### 3.1.2 Changes in the social environment

The main ideas of poverty control in China can be summarized as: in the early stage of development with relatively weak economic strength, emphasis is placed on solving the problem of resource-based poverty caused by widespread economic backwardness. (24) In the middle and later stages of development with relatively strong economic strength, efforts should be made to solve the problem of developmental poverty caused by relative welfare deprivation. Health poverty alleviation is a kind of development model for vulnerable groups in poor areas under the guidance of the concept of inclusive development. Its ultimate purpose is to pursue the growth of public welfare and achieve social equity and just expansion, featured with strong policy pertinence. The historical progress of China's health poverty alleviation policy exhibits the change of value concept: from the efficiency value orientation focusing on food and clothing and income issues to the fairness value orientation focusing on development and quality issues.

### 3.2 Meso level: path dependence

Historical institutionalism holds that institutional change is affected by learning effect, coordination effect and adaptive expectation, showing the characteristics of path dependence. Health poverty alleviation policies continue to develop under the requirements of the policy environment, but their changes are also affected by path dependence, revealing some signs of self-maintenance and reinforcement.

#### 3.2.1 Coordination effect

In the historical evolution, the national health poverty alleviation policy has formed a coordination effect of multi-system convergence, i.e., being coordinated with the basic medical insurance system, the reform of the medical and health system, and the incentive mechanism for health talents. The state has issued a number of supporting documents to coordinate and supplement the health poverty alleviation policy system. This coordination system has become an important path dependence of health poverty alleviation policies.

#### 3.2.2 Adaptive expectations

The development of China's health poverty alleviation policy has traces to follow, and the relevant goals proposed at all stages of development are subject to long-term adaptive institutional arrangements. In the embryonic and development stage of policies, various practices in the field of health poverty alleviation are paving the way for the proposal of health poverty alleviation projects, which are basically in line with the expected policy effects, and constantly strengthen the willingness of the Government to further improve health poverty alleviation. Consequently, adaptive expectations promoted the continuation and improvement of health poverty alleviation policies.

### 3.3 Micro level: subject interaction

#### 3.3.1 Central government

Health poverty alleviation projects are government-led policy public goods. The upgrading of policy formulation with the text content objectively reflects the strong catering of the policy product supply to social needs. The central government plays a leading role in all stages of health poverty alleviation policies by way of policy promulgation and financial support. At the same time, through the promulgation of policies and financial support, the government leads the development direction of such policies and therefore consolidates its leading position in health poverty alleviation.

#### 3.3.2 Social forces

In the last 30-odd years of poverty alleviation and development work, social forces have played a great role (25). It is worth mentioning that such work has been actively supported by lots of enthusiastic participants including authorities, enterprises and individuals. They have made obvious progress in the prevention and treatment of endemic diseases, congenital heart disease, and children's amblyopia. That's truly remarkable.

#### 3.3.3 Individual poor persons

The goal of interest pursued by poor groups is to improve their own poverty situation. In respond to the changes in health poverty alleviation policies, the role of poor groups is also constantly changing. In the embryonic stage of policies, poor groups were passive recipients, who gave little information feedback on the formulation and implementation of health poverty alleviation policies. In the period of policy formation and development, special poor groups received more attention. Thanks to the establishment of a new rural cooperative medical insurance system, great incentives and guarantees are provided for solving the problem of poor people who may suffer from falling into poverty or returning to poverty as a result of illness (26).

## 4 Conclusions and prospects

From the theoretical perspective of historical institutionalism, this paper elaborated China's health poverty alleviation policies. Combining historical experience induction and logical analysis, a targeted framework of dynamic mechanism analysis called “macrostructure—meso system—micro actor” was constructed. This paper also looked in the historical evolution of China's health poverty alleviation policy and discussed the dynamic mechanism of its change. It is foreseeable that the future development of health poverty alleviation policies will mainly focus on the following points:

- Improve the accessibility and effectiveness of health services: Most of the rural poor live in remote areas blocked in transportation. Improving the accessibility and effectiveness of health services means enabling poor people to see a doctor nearby and receive convenient medical and health services in a timely manner. Nonetheless, how to do is the key step to the success of health poverty alleviation. To this end, the ongoing work to be: First, put in more efforts in the construction of the medical and health service system in poor areas. The second is to implement one-on-one assistance between national tertiary hospitals and counties in contiguous poverty-stricken areas and county-level hospitals in key counties for poverty alleviation and development. The third is to invest more in the comprehensive training of talents.
- Encourage more participation of social forces in health poverty alleviation: Social forces have played an important role in poverty alleviation and development, and their participation should be further encouraged. Private enterprises, social organizations and individuals have not only paid close attention to, but also actively participated in China's poverty alleviation and development work. To give play to such a role of social forces, the following four points should be highlighted: First, do a good job in basic work, and clarify and accurately understand the causes and types of diseases. Second, provide an effective and stable docking platform between national, provincial, municipal, township and other relevant organizations to satisfy the needs of the people. Third, fully mobilize new social forces to participate in health poverty alleviation projects. Fourth, make full use of the functions of socio-professional institutions.
- Invest more in the construction of medical and health personnel in poverty-stricken areas: First, improve the professional level of existing talents. Second, cultivate local talents who may be retained, stabilized, and willing to take root. According to different situations, formulate different training orders and send talents out for training. After obtaining the qualification of assistant doctor, medical practitioner or general practitioner, better health services to the local people can be expected. Third, improve the remuneration of medical and health personnel in poor areas, and let them stay out of affection and attractive salary package. For example, while poor towns should build swing houses, preferential treatment should be given in terms of salaries and subsidies that are favorable for the retention of professionals who have feelings for poor areas and are willing to dedicate. Lots of jobs should be done to solve the problem of medical and health personnel in poor areas through multiple channels and methods.

## Data availability statement

The original contributions presented in the study are included in the article/supplementary material, further inquiries can be directed to the corresponding author.

## Author contributions

LX: Investigation, Writing – original draft, Formal analysis. YC: Methodology, Writing – review & editing. JY: Data curation, Writing – review & editing. XY: Funding acquisition, Investigation.
